# Modeling the potential impact of pre-exposure prophylaxis for HIV among men who have sex with men in Cameroon

**DOI:** 10.1186/s12879-022-07738-z

**Published:** 2022-09-26

**Authors:** Carrie E. Lyons, Owen J. Stokes-Cawley, Anna Simkin, Anna L. Bowring, Iliassou Mfochive Njindam, Oudou Njoya, Anne Zoung-Kanyi Bissek, Ubald Tamoufe, Sandra Georges, Florence Zeh Kakanou, Gnilane Turpin, Daniel Levitt, Serge Clotaire Billong, Sharmistha Mishra, Stefan Baral

**Affiliations:** 1 Department of Epidemiology, Johns Hopkins School of Public Health, 615 N Wolfe St, Baltimore, MD 21205 USA; 2 Centre for Urban Health Solutions, Li Ka Shing Knowledge Institute, St. Michael’s Hospital, University of Toronto, Toronto, Canada; 3 Department of Internal Medicine and Specialties, Faculty of Medicine and Biomedical Sciences, University of Yaoundé I, Yaoundé, Cameroon; 4 Department of Internal Medicine and Specialties, Faculty of Medicine and Biomedical Sciences, University of Yaoundé I, Yaoundé, Cameroon; 5 Division of Operations Research, Ministry of Health, Yaoundé, Cameroon; 6 Metabiota, Yaounde, Cameroon; 7 Johns Hopkins Cameroon Program, Yaounde, Cameroon; 8 CARE Cameroon, Yaoundé, Cameroon; 9 Directorate of Epidemic, Pandemic and Disease Control, Ministry of Public Health, Yaoundé, Cameroon; 10 CARE USA, New York, NY USA; 11 Department of Public Health, Faculty of Medicine and Biomedical Sciences, University of Yaoundé I, Yaoundé, Cameroon; 12 Central Technical Group, National AIDS Control Committee, Yaoundé, Cameroon

**Keywords:** Human immunodeficiency virus, Mathematical modeling, Pre-exposure prophylaxis, Men who have sex with men, Cameroon

## Abstract

**Background:**

Men who have sex with men (MSM) are consistently burdened by HIV at higher levels than other adults. While HIV prevention programs for MSM are growing in coverage and quality, HIV incidence remains high. In response, pre-exposure prophylaxis (PrEP) was introduced in 2019 to support HIV risk reduction among MSM in Cameroon. Understanding how PrEP initiation programs will change the HIV prevalence among MSM in Cameroon is important to developing effective programs.

**Methods:**

This study uses a mathematical model to simulate population-level HIV transmission among MSM in the cities of Yaoundé and Douala, Cameroon. PrEP is incorporated into the model at rates that equal 25%, 50%, or 75% coverage after twenty years to assess the potential effects on HIV prevalence among MSM, requiring annual initiation rates of 2.5%, 6.8%, and 17.2% for Yaoundé and 2.2%, 5.6%, and 13.4% for Douala, respectively. The data utilized for this model are from a cross sectional study which recruited MSM through respondent-driven sampling of MSM in two major cities in Cameroon: Yaoundé and Douala.

**Results:**

The model estimated an HIV prevalence of 43.2% among MSM, annual HIV diagnoses of 300 per 10,000 MSM and antiretroviral therapy (ART) coverage of 53.9% in Yaoundé. In Douala, estimated prevalence is 26.5% among MSM, 167 per 10,000 MSM annual diagnoses and ART coverage of 72.0%. Standalone PrEP interventions aimed at 50% coverage at the end of a 20-year program would reduce the prevalence from 43.2% to 35.4% in Yaoundé and from 26.5 to 20.1% in Douala. Combining PrEP with a 10% increase in HIV testing would decrease the number of MSM living with HIV and unaware of their status from 9.8 to 6.0% in Yaoundé and from 8.7 to 4.6% in Douala.

**Conclusions:**

PrEP would be beneficial in reducing prevalence even at varying initiation and coverage levels. Combination of PrEP and increased HIV testing further decreased the number of undiagnosed MSM. This study supports the utility of implementing PrEP as part of comprehensive HIV prevention programming among MSM in Cameroon.

**Supplementary Information:**

The online version contains supplementary material available at 10.1186/s12879-022-07738-z.

## Background

The Human Immunodeficiency Virus (HIV) pandemic continues to expand among men who have sex with men (MSM), despite declining HIV incidence among other populations [1–3]. Studies from high-income as well as low- and middle-income countries suggest that similar biological, network, and structural determinants heighten HIV risks among MSM around the world [4, 5]. HIV prevalence among MSM remains high in many regions of the world including Sub-Saharan Africa with prevalence estimates ranging from 4–33% [6]. In concentrated epidemics, such as across Africa, MSM are 3.8 times as likely to be living with HIV than other adult men [4, 7]. Importantly, this burden is also disproportionate even within generalized epidemics [8]. Data estimating HIV incidence among MSM are limited, however among those available estimates are as high as approximately 14 per 100 person-years [9].

Notably, HIV epidemics among MSM continue to grow around the world, in part due to biologic factors such as the high HIV transmission probability of condomless receptive anal intercourse with a serodiscordant and viremic sexual partner, which is over ten times higher than that of condomless vaginal sex [10, 11]. While the study of individual HIV risks has often been the focus of studies among MSM, network or social determinants as well as structural determinants potentiate HIV risks among MSM. Social determinants such as social cohesion and support, as well as structural barriers such as stigmas, laws, policies, and organizational programs all influence access to healthcare services and interventions [4, 5].

Significant and frequent unmet needs for HIV prevention and treatment services among MSM serve as social and structural barriers to the uptake and provision of HIV prevention continue to play a major part in perpetuating these HIV epidemics [4]. The dynamic of ignoring the needs of MSM was documented early in the HIV pandemic with limited study and development of HIV prevention strategies [12, 13]. MSM consistently report high levels of stigma which has been shown to increase HIV risks through limited engagement and access to interventions [4]. In 2022, stigma among MSM remains high in many regions, including across Sub-Saharan Africa [4, 5, 14], where individual and structural stigmas have hindered engagement and access to interventions. Moreover, these stigmas and criminalization of same-sex practices also limit disclosure of same-sex practices to healthcare providers which further limits the provision of appropriate services [4, 5, 14]. For example, one study found that only less than a quarter of MSM disclosed their sexual practices to healthcare professionals in Botswana, whereas less than one in ten disclosed in Malawi [15, 16]. Stigma remains a barrier to healthcare uptake, access, and quality, especially in settings which criminalize same sex behaviors, such as in the Central African country of Cameroon. Although stigma is not the only barrier to HIV prevention among MSM, it continues to limit the impact of HIV prevention and treatment technologies, medications, and strategies and its elimination is a key target for UNAIDS. The availability and access to multiple and diverse HIV prevention options allow MSM to select prevention methods which are appropriate for their lifestyles, especially in context of barriers to healthcare.

Biomedicial prevention services such as Pre-Exposure Prophylaxis (PrEP) have provided new prevention opportunities to limit acquisition and onward transmission of HIV. Over the last decade, PrEP has shown to be safe and effective in preventing HIV among individuals at an increased risk of infection [17, 18]. And in the context of sustained and growing HIV epidemics among MSM, PrEP has emerged as an HIV prevention tool that can effectively decrease HIV incidence among MSM around the world [17–19]. While trials and demonstration studies have established individual-level efficacy and effectiveness of PrEP [18, 20], mathematical models have allowed for estimation of potential population-level impact of PrEP. For example, a modeling study of the effects of PrEP on MSM in Toronto, Canada, found that the use of oral PrEP led to reductions in incident HIV infections, especially when high-risk MSM are engaged with care such as HIV testing and prevention services [21]. Another study modeling PrEP among MSM and transgender women in Peru found that low levels of PrEP coverage among high priority individuals could be cost-effective despite requiring a large financial backing to implement [22].

As of 2019, only ten countries across Sub-Saharan Africa have approved Truvada (PrEP) or its generic form [23, 24]. To date, most countries have yet to scale up PrEP including in countries across West and Central Africa. In Cameroon specifically, PrEP has yet to be fully scaled in a setting where the HIV prevalence among adults is around 5% with estimates of more than a third of MSM living with HIV [25]. Scale up of PrEP initiated in early 2021 in six cities with high concentrated key populations including Bafoussam, Ngaoundéré, Kribi, Bertoua, Bamenda and Buea. Although PrEP has not yet been widely available in Cameroon, the new national HIV strategic plan has included PrEP as an intervention for HIV prevention for MSM and female sex workers [26]. Therefore, modeling the potential impact of PrEP on HIV prevalence in the two largest cities in Cameroon can help inform the level of implementation needed to achieve prevention outcomes. Additionally, modeling PrEP use among MSM may help inform the strategy for scaling up PrEP in Cameroon and other countries in the region. To support PrEP implementation in Cameroon, this study will model the transmission characteristics of HIV among MSM in Cameroon and estimate the impact of implementing varied PrEP interventions.

## Methods

### Model

**Fig. 1:**
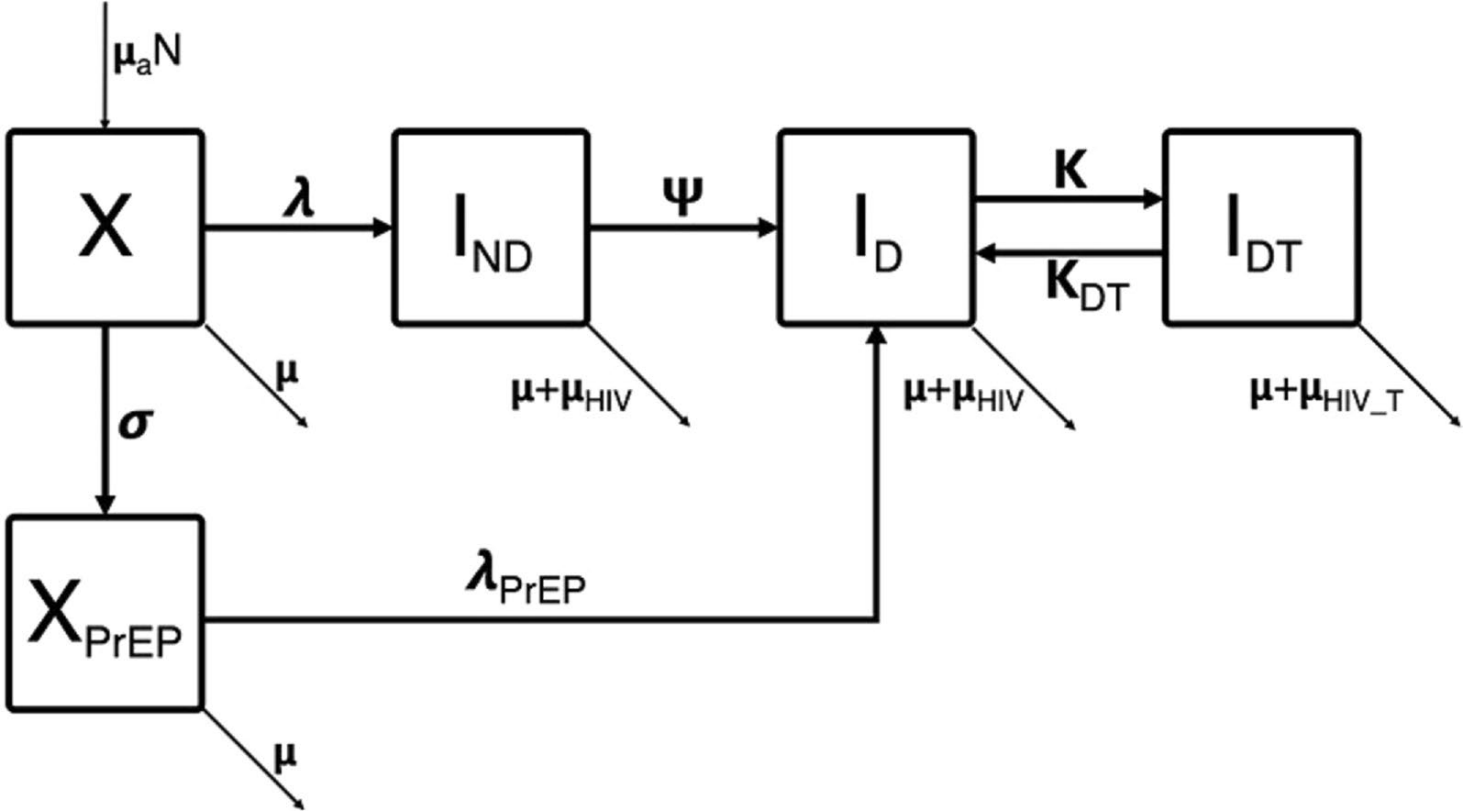
Model figure. The compartments are: susceptible to infection (X), susceptible while on PrEP (X_PrEP_), infected but not diagnosed (I_ND_), infected and diagnosed but not on treatment (I_D_), and infected, diagnosed and on treatment (I_DT_). The rates of exchange between compartments are: the force of infection (λ), force of infection while on PrEP (λ_PrEP_), rate of PrEP initiation (σ), rate of HIV testing (ψ), rate of ART initiation (κ), and rate of ART dropout (κ_DT_), Entry and exit rate not due to HIV (μ), exit rate while infected (μ_HIV_), and exit rate while infected and on treatment (μ_HIV_T_). Individuals who are on PrEP (X_PrEP_) who become infected become aware of their HIV status within one year and therefore move to the diagnosed (I_D_) compartment

This study used a mathematical model to understand the transmission characteristics of HIV among MSM in two major cities in Cameroon and to estimate the impact of implementing varied PrEP interventions. The mathematical model used here has been previously described [27, 28]. The model was created to study and estimate the impact of PrEP on transmission of HIV among MSM. Previous applications have focused on cities in Canada, but the parameters and population dynamics and demographics have been changed to allow for appropriate use in Cameroon. The model is intended to simulate HIV transmission among MSM in Cameroon with the ability to incorporate the impact of PrEP intervention in order to estimate the prevalence of HIV among MSM based on a variable PrEP initiation rate. This is a deterministic, compartmental model with 5 compartments; susceptible to infection (X), susceptible while on PrEP (X_PrEP_), infected but not diagnosed (I_ND_), infected and diagnosed but not on treatment (I_D_), and infected, diagnosed and on treatment (I_DT_). The model is represented in Fig. 1.

The movement through compartments is determined by the rates at which people become infected, find out they are positive through testing, initiate or stop treatment, and initiate PrEP. These are represented by the force of infection (λ), force of infection while on PrEP (λ_PrEP_), rate of PrEP initiation (σ), rate of HIV testing (ψ), rate of ART initiation (κ), and rate of ART dropout (κ_DT_), Entry and exit rate not due to HIV (μ), exit rate while infected (μ_HIV_), and exit rate while infected and on treatment (μ_HIV_T_). Both of the force of infection parameters (λ and λ_PrEP_) are based on two activity levels using the number of concurrent partners, the number of sex acts per partnership, and the per act transmission probability, outlined in Table 1. One key assumption made was that individuals on PrEP who became infected were aware of their HIV status within one year and therefore moved straight to the diagnosed compartment (I_D_). This assumption exists because PrEP initiation requires HIV screening. If an individual is HIV-positive and unaware, they will screen prior to PrEP initiation and become diagnosed. Another assumption made was that this population did not actively participate in serosorting and therefore exhibited proportional mixing. Additionally, there was proportional mixing between the high and low activity group as well.

**Table 1:** Model parameters. The first five parameters: proportion of high activity group, ratio of number of partnerships of high-activity group to the low-activity group, transmission risk of receptive acts, HIV testing rate per year and ART initiation rate per year, all used ranges in the calibration. The latter parameters used point estimates based on the sources

Model		Range or value		Final model		
Parameter	Symbol	Yaounde	Douala	Yaounde	Douala	Source
Proportion of high activity group	N_H_/(N_H_ + N_L_)	[0.073, 0.196]	[0.124, 0.217]	0.178	0.193	[25, 29]
Ratio of number of partnerships high-activity group to low-activity group	C_H_/C_L_	[3.977, 6.792]	[4.805, 6.636]	6.31	6.16	[25, 29]
Condom use proportion,	pc_11_	[0.418, 0.707]	[0.467, 0.714]	0.451	0.508	[29]
negative-negative						
HIV testing rate (per year)	$\psi$	[0.61, 0.69]	[0.65, 0.67]	0.649	0.661	[29]
ART initiation rate (per year)	k	[0.07, 0.51]	[0.08, 0.79]	0.241	0.532	[29]
ART dropout rate	k_D_	0.15	0.15	0.15	0.15	[29]
Proportion of sex acts insertive	α	0.5	0.5	0.5	0.5	[29]
Proportion of sex acts receptive	1-α	0.5	0.5	0.5	0.5	[29]
Number of concurrent partners, low activity group (per person per year)	C_L_	4	5	4	5	[29]
Condom use odds ratio,	OR_pc_12_	2.203	2.203	2.203	2.203	[29]
positive–negative						
Proportion of viral suppression among ART users	T_ART_	0.89	0.91	0.89	0.91	[29]
Transmission risk, insertive acts	ß_i_	0.0011	0.0011	0.0011	0.0011	[46]
Transmission risk, receptive acts	ß_r_	0.0073	0.0073	0.0073	0.0073	[46]
Annual number of sex acts per partnership	Nsa	20	16	20	16	[29]
Condom efficacy	d_condom_	80%	80%	80%	80%	[47]
PrEP efficacy	Ω	44%	44%	44%	44%	[18]
Entry/exit rate	m	0.026	0.026	0.026	0.026	[29, 48]
Exit rate due to HIV mortality	m_HIV_	0.076	0.076	0.076	0.076	[49]
Exit rate due to HIV mortality while on ART	m_HIV_T_	0.031	0.031	0.031	0.031	[49]

### Parameters

The model is parameterized by data collected through an Integrated Biological and Behavioral Surveillance (IBBS) survey among men who have sex with men in Cameroon [29]. This cross-sectional survey was conducted in 2016 and in five major urban centers in Cameroon, Yaoundé, Douala, Bertoua, Bamenda, and Kribi. Data from the two largest cities, Yaoundé and Douala, are used for this model. Participants were recruited through respondent driven sampling (RDS) and trained interviewers gathered data through a behavioral questionnaire and serological testing. Ethical approval and administrative clearance were obtained from the Cameroonian National Research Ethics Committee (reference 2015/05/591/CE/CNERSH/SP and 2016/06/782/CE/CNERSH/SP) and Ministry of Public Health (reference 631 2315), respectively. Secondary data analysis of these data was approved by Johns Hopkins University (JHU) Bloomberg School of Public Health Institutional Review Board under IRB00007006. When data was not available from this study we used data from similar areas throughout Central and Eastern Africa, summarized in Table 1.

In order to accurately parameterize the model, we used ranges from the IBBS 2016 survey to fit five of the model parameters and the three outcome parameters. The rest of the parameters were fit with point estimates for efficiency through IBBS 2016 or similar literature. All model parameters are outlined in Table 1. The outcome parameters were based on prevalence, rate of new diagnoses (per 100,000 MSM) and ART coverage over the past 10 years. Based on the IBBS and other sources (referenced in Table 1), the outcome parameter ranges were found to be 35.7–53.2% prevalence, 98 to 1405 diagnoses per 10,000 MSM, and 42.9–66.7% ART coverage in Yaoundé. In Douala, the ranges were found to be 19.1–31.9% prevalence, 33–360 annual diagnoses per 10,000 MSM and 57.1–90.6% ART coverage.

### Calibration

Calibration of this model was done using Latin Hypercube Sampling (LH) for the five model parameters with ranges in order to optimize the results from this model. Using LH, each of the five parameters were split into six equal sized bins. Every possible bin combination was used to create a sample of simulated epidemics, for a total of 6^5^ simulations. For every simulated epidemic, the value within each bin was sampled randomly within the ranges of that bin. The model was run for 100 years to allow it to converge. If the results of the epidemic fell within the ranges of all three outcome parameters (prevalence, new diagnoses, and ART coverage), they were kept for further analysis. Of the simulated epidemics that fit all outcome parameters, maximum likelihood was tested for each outcome parameter. The maximum likelihood values for each outcome parameter were combined in even weights for each simulated epidemic to form a cumulative maximum likelihood value. The 100 epidemics with the highest cumulative maximum likelihood were used to parameterize the original compartmental model.

### Intervention

After calibration, the original deterministic model was run. First, it was run without any PrEP intervention to create a baseline from which the effects of PrEP could be drawn. The effects were assessed by the change in prevalence and change in proportion undiagnosed. These were based on different PrEP coverage levels over a 20-year intervention and the additional benefit of increased HIV testing due to PrEP intervention. Data calculations for the model and outcome parameters were done using STATA 14.2 (College Station, Texas). The model was run and calibrated using MATLAB R2017b (9.3.0.713579) maci64.

### Patient and public involvement

This study uses data from the IBBS survey conducted in Yaoundé, Douala, Bertoua, Bamenda, and Kribi among men who have sex with men in Cameroon, as described previously[29]. This was a cross-sectional, respondent-driven sampling study performed in 2016. The IBBS design, survey tool, and recruitment strategies were informed by input and participation from MSM and community leaders. The implementation of this study was done in partnership with Care Cameroon. Dissemination of results from this study will be done under the leadership of Care Cameroon, Metabiota, and the networks of MSM in Cameroon.

## Results

### Calibration

For Yaoundé and Douala, each model was calibrated over a 100-year timeframe, allowing it to converge (Additional file 1: Fig. S1). From the initial 6^5^ simulations, the results that were within all three outcome parameter ranges were analyzed using maximum likelihood. From the maximum likelihood estimates, the top 100 epidemics were further analyzed. The median of these 100 maximum likelihood results are summarized in Table 1. The median model for Yaoundé resulted in a prevalence of 43.2% among MSM, new annual diagnoses of 300 per 10,000 MSM, and ART coverage of 53.9%, while the median model from Douala found a prevalence of 26.5% among MSM, new annual diagnoses of 167 per 10,000 MSM and ART coverage of 72.0%.

### Intervention

**Fig. 2:**
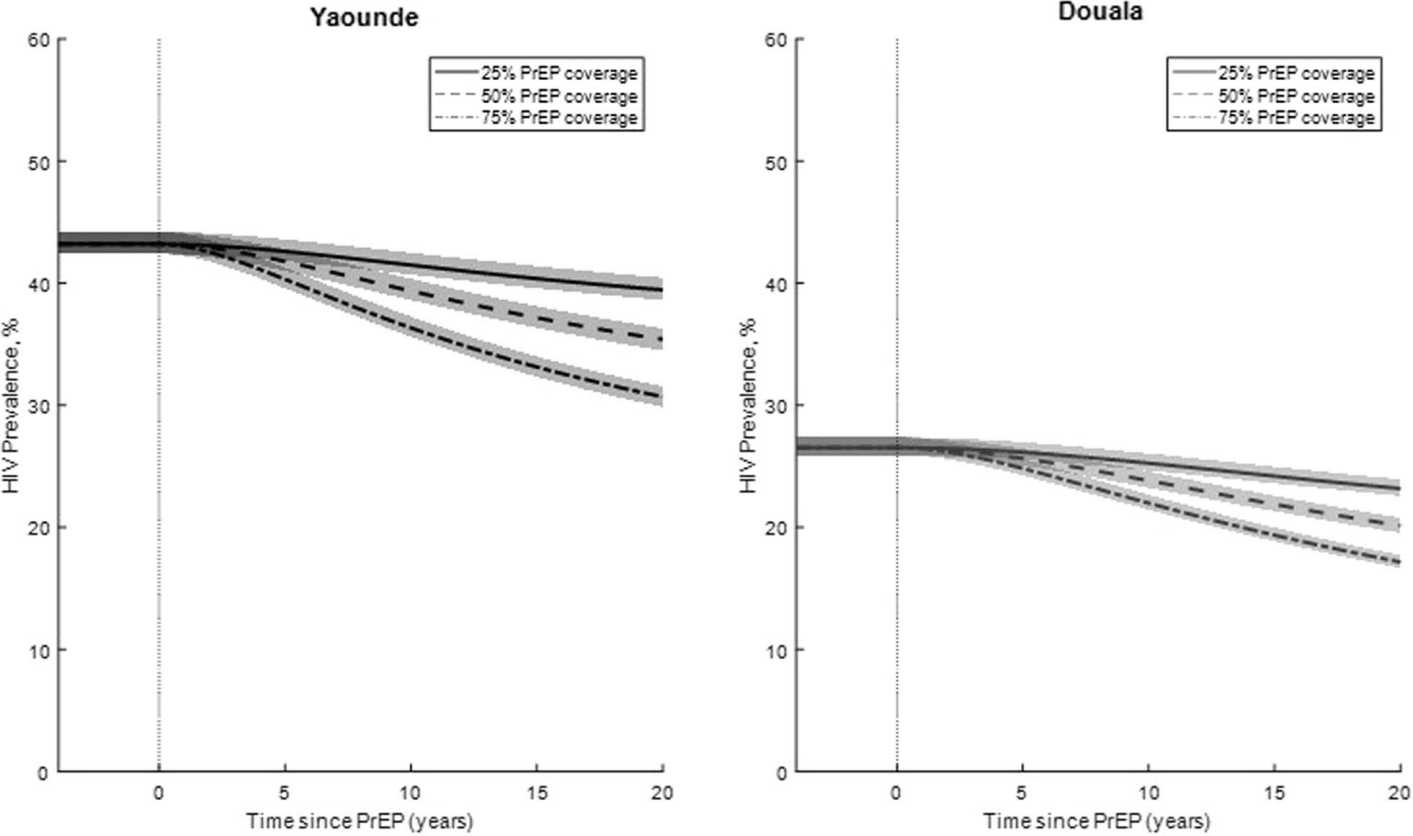
Projected prevalence. HIV prevalence in Yaounde and Douala based on a varied 20-year PrEP coverage programs aimed at 25%, 50% and 75% coverage at the end of the program. Lines represent the median epidemic while the shaded area represents the area between the 25th and 75th percentile of the 100 best fit epidemics

After running the model for 100 years, a standalone PrEP intervention was added to the models. PrEP was added at a set rate per year in order to achieve a PrEP coverage of 25%, 50% or 75% at the end of a 20-year program. Figure 2 shows the prevalence of HIV in Yaoundé and Douala following a PrEP intervention program with varying PrEP coverage estimates. The figure shows the change in HIV prevalence since the start of the intervention in year 0. In Yaoundé, a PrEP initiation rate of 6.8% of tested MSM per year would need to go on PrEP in order to achieve a coverage of 50% at the end of a 20-year program. PrEP initiation would need to be 2.5% or 17.2% of tested MSM per year in order to achieve coverage of 25% and 75% PrEP coverage after 20 years, respectively. In this model, tested MSM represents individuals who are already in contact with the healthcare system and who are HIV negative. MSM experience barriers to engaging in healthcare, especially in settings with high levels of stigma. Therefore, MSM in contact with the healthcare system represents a population that might be more willing to seek care and enroll in a PrEP program. While HIV negative MSM overall represents the target population for PrEP, the tested MSM population represents a more realistic target to engage in PrEP.

Implementing a 20-year program that would achieve 50% PrEP coverage would result in a change in prevalence from 43.2% to 35.4% (Fig. 2). Programs that would achieve 25% and 75% coverage would result in a prevalence of 39.4% and 30.7%, respectively (Fig. 2).

In Douala, a program that has a consistent PrEP initiation of 5.6% of tested MSM annually would result in a PrEP coverage of 50% after 20 years. Similar length programs with PrEP initiation of 2.2% and 13.4% would result in PrEP coverage of 25% and 75%, respectively. The prevalence in Douala would decrease from 26.5% to 20.1% following a 20-year program that would result in 50% PrEP coverage (Fig. 2). Similar programs resulting in 25% and 75% PrEP coverage would result in a prevalence of 23.2% and 17.2%, respectively (Fig. 2).

**Fig. 3:**
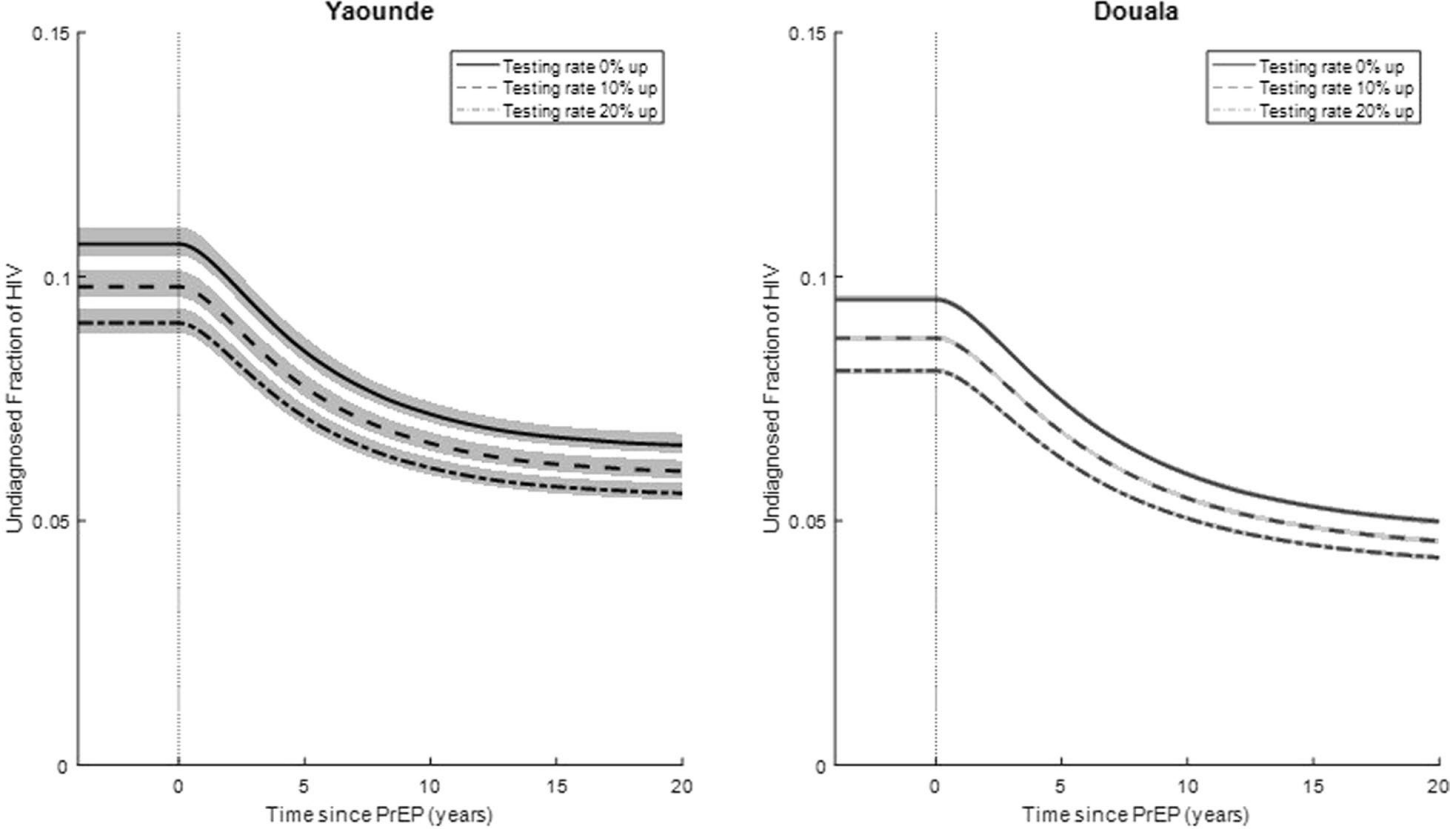
Undiagnosed proportion. The percent of infected MSM who are undiagnosed out of total infected in Yaounde and Douala. PrEP intervention was introduced at time 0 in addition to increased testing rate among MSM by 10% and 20% from baseline (65/100 person-years). Lines represent the median epidemic while the shaded area represents the area between the 25th and 75th percentile of the 100 best fit epidemics

In Yaoundé under current HIV testing conditions, a 20-year PrEP intervention aimed at 50% PrEP coverage would result in the fraction of HIV positive MSM that are undiagnosed decreasing from 10.7% to 6.6% over the course of the intervention with a baseline HIV testing rate of 65/100 person-years (Fig. 3). In a scenario where HIV testing is increased 10% over current rates, an intervention aimed at 50% PrEP coverage would decrease the proportion of undiagnosed HIV positive MSM from 9.8% to 6.0% (Fig. 3). In a scenario where the HIV testing rate is 20% increased compared to current levels, the proportion of undiagnosed MSM would decrease from 9.1% to 5.6% (Fig. 3).

In Douala, a 20-year program achieving 50% PrEP coverage would decrease the proportion of HIV positive MSM that are undiagnosed from 9.5% to 5.0% with the baseline HIV testing rate (Fig. 3). An increased testing rate of 10% would result in a change from 8.7% to 4.6%. A PrEP program combined with a 20% increase in HIV testing rate would change the proportion of undiagnosed MSM from 8.1% to 4.3% (Fig. 3).

## Discussion

Encouragingly, the results from these models reinforce the potential impact of PrEP in Cameroon with the greatest benefits measured by decreased HIV prevalence coming when PrEP coverage levels are optimized. Increased testing associated with PrEP implementation would result in significant decreases in undiagnosed HIV infections facilitating linkage to treatment programs. Moreover, the models highlight the potential impact of PrEP in Cameroon even with limited coverage supporting the decision to implement PrEP in Cameroon for MSM.

PrEP was scaled to reach different levels of coverage at the conclusion of a 20-year intervention: 25%, 50% and 75% coverage. Challenges, such as knowledge and attitude towards PrEP, may act as barriers to implementing large PrEP interventions, making effective but modest interventions very appealing. A study in South Africa found that the vast majority of MSM surveyed were willing to take PrEP[30]. Positive perceptions of PrEP are likely to increase among MSM as exposure increases, but implementation generally achieves limited coverage in the earlier phases of these programs. Given the benefits of PrEP even at low levels of coverage in Cameroon, the program could start even with modest initiation rates and then scale with increasing demand. Notably, PrEP programs also include more frequent HIV and potentially STI tests and more general healthcare engagement so may not be for everyone[30]. Consequently, it remains crucial to implement PrEP as part of a comprehensive program that includes the provision of commodities such as condoms and condom compatible lubricants.

The standalone intervention from the Yaoundé and Douala models reduced prevalence of HIV, but a standalone intervention is not expected to end HIV transmission[31]. The data presented here and elsewhere demonstrate the importance of effective integration of HIV testing with PrEP implementation given this is an HIV-status dependent intervention[32]. The model results indicate that a 10% increase in testing among all individuals could decrease HIV prevalence and further prevent new infections when combined with a PrEP intervention. Individuals who are unaware of their infections are at significant risk of onward HIV transmission given higher rates of condomless sex with people at risk of HIV acquisition [33–36]. Routine HIV testing remains a critical component of HIV prevention and a recommendation of CDC in order to reduce the proportion of people living with HIV who are unaware of their status [37]. These data are consistent with a modeling study among MSM in South Africa which showed that reducing the number of individuals that did not receive an HIV test each year from 1/3 to 1/6 of the population resulted in around a 5% increase in prevented HIV infections [38]. While PrEP and increased HIV testing appear to be necessary to reduce HIV risks, they are not sufficient and require additional services including community-delivered prevention outreach services, appropriate education, and early access to ART for those living with HIV[39].

In addition to the immediate health benefits of preventing HIV, some researchers have found health benefits associated with PrEP that are rarely studied. For example, a study from Seattle found that using PrEP reduced anxiety and stress regarding their sexuality [40]. PrEP is a prevention method which allows individuals to use this biomedicial intervention during specific periods of their life where it may needed. Adherence to PrEP during periods of high-risk sexual activity is important to its effectiveness, [41] but while individuals are in periods of their life with low risk for HIV acquisition, continuation of PrEP may not be necessary. This is in contrast to antiretroviral therapy for treatment among people living with HIV, in which adherence is needed through their entire life for viral suppression.

There were several limitations in this study which should be considered. The primary outcome of this study is HIV prevalence which may be a delayed indicator of the impact of PrEP compared to HIV incidence. Prevalence may also be subject to other HIV outcomes such as mortality among MSM living with HIV, although in this model mortality was held constant over time in order to isolate the impact of PrEP. This model did not incorporate implementation strategies based on risk heterogeneity among MSM. Therefore, it may be possible to achieve equivalent impacts at lower coverage if risk differences among MSM are incorporated into PrEP implementation strategies [42]. Understanding and incorporating risk heterogeneity among MSM to inform PrEP implementation may be an important next step. Importantly, there are limited data within Cameroon, especially among MSM, and some of the model parameters were estimated from settings from other settings across West and Central Africa. Second, a large proportion of these data was based on a study that used respondent-driven-sampling to attract participants. The underlying mathematical model based several parameters on the adult population and the use of health clinics as opposed to a research study. This may bias results towards individuals that are living with HIV. This model used a combination of the baseline entry rate by µ and the additional entry rate that reflects population growth (µ0). This results in a population that is balanced in terms of high activity and low activity size throughout the timeframe of the model. This may not accurately represent the entering cohort because HIV-related deaths are more likely to occur among higher-risk and sexually active MSM as a result of higher activity and therefore higher HIV prevalence. However, the research questions here did not involve comparing interventions targeting different activity groups of MSM specifically, and instead, focused on the impact among all MSM where these differences are insignificant. Several assumptions are made in this model.

This model assumes MSM are aware of their HIV positive status within one year and the model is unable to represent undiagnosed HIV infection while on PrEP given the model steps are in one year increments. Therefore, the results do not account for MSM who are undiagnosed, on PrEP, and acquired HIV. In this specific model, entry on PrEP is unidirectional and does not incorporate discontinuation, which is likely to occur. Lastly, PrEP adherence and persistence (based on oral daily PrEP) is not taken into account in this model and an important consideration in implementation and effectiveness.

Though there have been incidence reductions overall in Cameroon, the HIV epidemics among MSM including young MSM appear to be sustained and even growing in the context of programs relying on condom distribution and HIV education programs for MSM. Therefore, additional strategies for prevention are needed to achieve progress in addressing the HIV epidemic among MSM in Cameroon. PrEP coverage in Cameroon is currently low, since programs only began to be implemented in 2019 alongside the inclusion of PrEP in the national strategic plan for HIV[26]. Given the high risk of HIV among MSM, PrEP represents an additional intervention to be included among a suite of biological, behavioural, and structural interventions. PrEP implementation for MSM in Cameroon will face several challenges similar to what has been observed in other settings since it represents a new intervention in a resource-constrained environment for a population that is affected by intersecting social and healthcare stigmas. However, lessons can be learned from other settings where PrEP scale up has already taken place [43]. For example, demand creation strategies as well as support for PrEP continuation in order to achieve the modelled levels of PrEP coverage may be needed. Additionally, PrEP options are expanding with the recently shown effectiveness of injectable PrEP as a method for prevention for those who prefer this method over oral PrEP [44, 45].

The results of the model presented here demonstrates the potential of PrEP to reduce HIV prevalence and also decrease the number of MSM who are unaware of their HIV status in Cameroon even with modest coverage during early implementation. Similar to other settings, implementation, acceptability, and uptake challenges can be overcome by allowing PrEP to complement existing interventions and facilitate a future where young MSM in Cameroon can have improved sexual health while minimizing HIV acquisitions risks. Importantly, this study may to inform PrEP implementation strategies in Cameroon, but also for other countries in West and Central Africa.

## Supplementary Information

**Additional file 1. Figure S1.** Calibration. Model structure.

## Data Availability

The datasets used and/or analysed during the current study are available from the corresponding author on reasonable request.

## Declarations

### Methods

All methods were carried out in accordance with relevant guidelines and regulations.
