# Supplementary material for: Modeling the potential impact of pre-exposure prophylaxis for HIV among men who have sex with men in Cameroon
Source: BMC Infect Dis. 2022 Sep 26;22:751. doi: 10.1186/s12879-022-07738-z (PMC9513877; doi:10.1186/s12879-022-07738-z)
Supplement: Supplementary file 1 — Additional file 1. Figure S1. Calibration. Model structure. [file 12879_2022_7738_MOESM1_ESM.docx]

**Additional file 1**


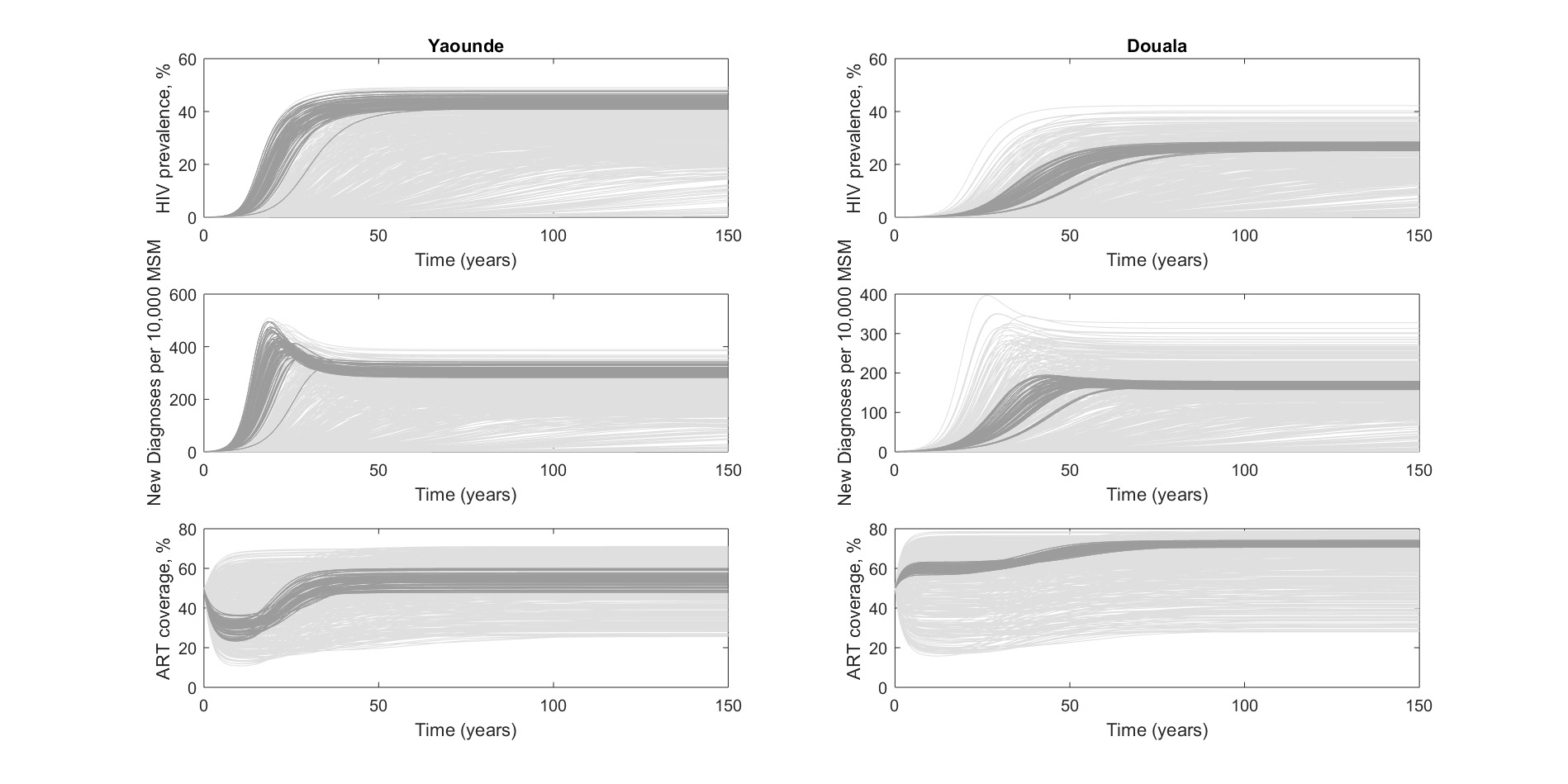


**Figure 1.** **Calibration.** Sampling from the calibration. Light grey lines are 500 simulations that did not satisfy all outcome parameters. Dark grey lines are the 100 best fit simulations. Not all simulations are represented on this figure.

1. **Model Structure**

This study adopted an HIV transmission dynamic model previously developed^1^. It is a deterministic, compartmental model for the simulation of HIV transmission, represented by a set of coupled ordinary differential equations, which are solved numerically using Euler integration implemented in MATLAB (R2017b).


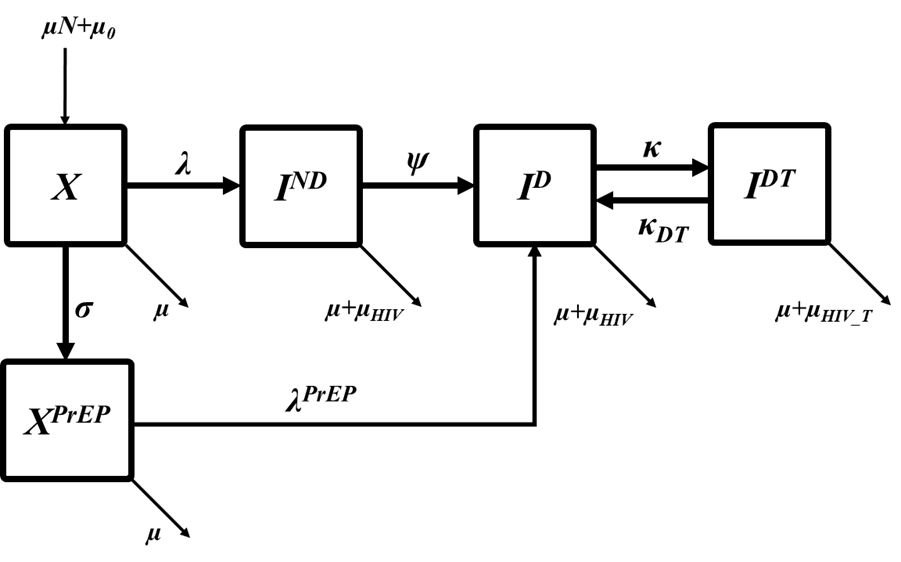


Figure A1. Flow diagram of natural history of infection and ART initiation. Susceptible persons (*X*) acquire HIV at the Force of Infection rate *λ_i_*, and live unaware of their HIV infection (*I^ND^*) and get tested at a rate of $\psi$ until they are diagnosed (*I^D^*), while those enrolled in PrEP (*X^PrEP^*), following HIV acquisition at rate *λ_i_*^PrEP^ become diagnosed directly following HIV infection (*I^D^*), due to high rate of HIV testing while on PrEP. HIV-infected individuals may also initiate ART, at rate *κ*, if they are diagnosed (*I^DT^*), while some of those on ART treatment may discontinue it at the rate *κ_DT_.*

State variables and transitions are shown in Figure A1.

The population is stratified into two activity groups, based on the number of partnerships per year:

- *low activity (i = 1)*
- *high activity (i = 2)*

Each state variable is based on the HIV status (serostatus) of the individuals:

- *susceptible (X_i_);*
- *susceptible on PrEP (X_i_^PrEP^);*
- *infected, undiagnosed (I_i_^ND^);*
- *infected, diagnosed (I_i_^D^);*
- *infected, diagnosed, on antiretroviral (ART) treatment (I_i_^DT^).*

For the purposes of mixing matrix definitions, the state variables can be grouped further based on the *perceived* serostatus, since the mixing preferences follow perceived serostatus rather than actual serostatus:

- *perceived uninfected/unknown (j = 1: X_i_, I_i_^ND^),*
- *diagnosed with HIV (j = 2: I_i_^D^, I_i_^DT^),*
- *uninfected on PrEP (j = 3, X_i_^PrEP^)*

HIV uninfected individuals enter into the susceptible compartment (*X_i_*) upon onset of sexual activity, at the baseline entry rate *µ*. The additional entry rate *µ_0_* reflects the population growth and is assumed to balance the HIV-related mortality.

$$\mu_{0}=\mu_{HIV}I^{ND}+\mu_{HIV}I^{D}+\mu_{HIV\_T}I^{DT}$$

The population exits the model at the rate *µ*, which reflects a combination of baseline mortality or cessation of sexual activity. However, the individuals living with HIV also exit via HIV-attributable mortality (*µ _HIV_* or *µ _HIV_T_*).

Individuals in the (*X_i_*) compartment can initiate Pre-exposure Prophylaxis (PrEP) at a rate *σ* which includes testing for HIV and uptake of PrEP, with transition into the *X_i_^PrEP^* compartment.

$$\sigma=\psi_{PrEP} PrEP\_uptake$$

Individuals in the *X_i_* compartment who acquire HIV infection transition into the *I_i_^ND^* compartment, which represents people living with undiagnosed HIV. Following HIV testing at a rate of *ψ*, individuals move into the *I_i_^D^* compartment (diagnosed with HIV, not on ART), after which they initiate antiretroviral treatment at a rate *κ* to move to compartment *I_i_^DT^* (diagnosed with HIV, on ART treatment). Some individuals discontinue ART at a rate *κ_DT_* and return to the *I_i_^D^* compartment.

The individuals in *X_i_^PrEP^* compartment, after acquiring HIV infection with the force of infection *λ_i_^PrEP^*, move directly into the *I_i_^D^* compartment, assuming that all individuals in *X_i_^PrEP^* undergo regular HIV testing, and the time spent with undiagnosed HIV is negligible for these individuals.

Details on model parameters and transition rates can be found in Table 1 (Main text) and Section 3 below.

***Model equations***

The mathematical model is comprised of the system of ordinary differential equations, where the dependent variables are the model state variables, and the independent variable is time.

1. Susceptible, not on PrEP.

$$\frac{dX_{i}\left( t \right)}{dt}=\mu N_{i}+\mu_{0}-\left( \lambda_{i}\left( t \right)+\sigma+\mu\right)X_{i}\left( t \right)$$

2. Susceptible, on PrEP.

$$\frac{dX_{i}^{PrEP}\left( t \right)}{dt}=\sigma X_{i}\left( t \right)-\left( \lambda_{i}^{PrEP}\left( t \right)+\mu\right)X_{i}^{PrEP}\left( t \right)$$

3. Infected, undiagnosed.

$$\frac{dI_{i}^{ND}\left( t \right)}{dt}=\lambda_{i}\left( t \right)X_{i}\left( t \right)-\left( \psi+\mu+\mu_{HIV} \right)I_{i}^{ND}\left( t \right)$$

4. Infected, diagnosed, not on ART treatment.

$$\frac{dI_{i}^{D}\left( t \right)}{dt}=\lambda_{i}^{PrEP}\left( t \right)X_{i}^{PrEP}\left( t \right)+\psi I_{i}^{ND}\left( t \right)+\kappa_{DT}I_{i}^{DT}\left( t \right)-\left( \kappa+\mu+\mu_{HIV} \right)I_{i}^{D}\left( t \right)\text{ }$$

5. Infected, diagnosed, on ART treatment.

$$\frac{dI_{i}^{DT}\left( t \right)}{dt}=\kappa I_{i}^{D}\left( t \right)-\left( \kappa_{DT}+\mu+\mu_{HIV\_T} \right)I_{i}^{DT}\left( t \right)\text{ }$$

The total population in our study is assumed to stay constant at any time:

$$N_{i}\left( t \right)=X_{i}\left( t \right)+ X_{i}^{PrEP}\left( t \right)+ I_{i}^{ND}\left( t \right)+ I_{i}^{D}\left( t \right)+ I_{i}^{DT}\left( t \right)\text{ }$$

1. **Force of Infection**

The force of infection *λ_i_* is the risk of acquiring HIV infection per susceptible individual. The force of infection depends on the following parameters: average number of partners per year (*C_i_*), probability of partnership between the individual (activity *i* and serostatus *j*) and the partner (activity *i*’ and sersostatus *j*’) as shown by the mixing matrices (*ρ_i,i’_* , *p_j,j’_*), and the transmission probability per partnership (*β_j,j’_*).

$$\lambda_{i}\left( t \right)=C_{i}\sum_{i^{'}=1}^{2} \sum_{j^{'}=1}^{3} \rho_{i,i'}p_{j,j^{'}}\beta_{j,j^{'}}\left( \frac{I_{i^{'}j^{'}}\left( t \right)}{N_{i^{'}j^{'}}\left( t \right)} \right)$$

The force of infection for those on PrEP is defined by the *λ_i_^PrEP^* term, where *ω* is PrEP effectiveness.

$$\lambda_{i}^{PrEP}\left( t \right)=(1-\omega)\lambda_{i}\left( t \right)$$

***Transmission risk***

The probability of transmission per partnership (*β*) depends on the biological probability of being infected during total number of sex acts per partnership per year, and the proportion of sex acts with condom (which depends on the type of partnership in the model). In general, transmission probability per partnership between susceptible person *j* and infected partner *j*’ can be defined as:

$$\beta_{j,j'}=\left( 1-{pc}_{j,j'} \right)\left( 1-\beta' \right){+pc}_{j,j'}\left( 1-{\beta'}_{c} \right)$$

Where

- *β*’ is the probability of *not being infected*, and (1 - *β*’) is the probability of *being infected* during sex acts without condom, per partnership, per year;
- *β*’_c_ is the probability of *not being infected*, and (1- *β*’_c_) is the probability of *being infected* during sex acts with condom, per partnership, per year;
- *pc_j,j’_* is the proportion of sex acts with condom for the partnership type *j,j’*.

The probability of *not being infected* per partnership per year depends on the biological transmission risk per one sex act, and the number of sex acts per partnership per year, *n_sa_* assumed to represent independent Bernoulli trials^2^:

$$\beta'=\left( 1-\beta_{ins} \right)^{\alpha_{j,j'}n_{sa}}\left( 1-\beta_{rec} \right)^{\left( 1-\alpha_{j,j'} \right)n_{sa}}$$

$${\beta'}_{c}=\left( 1-{\left( 1-d_{c} \right)\beta}_{ins} \right)^{\alpha_{j,j'}n_{sa}}\left( 1-{\left( 1-d_{c} \right)\beta}_{rec} \right)^{\left( 1-\alpha_{j,j'} \right)n_{sa}}$$

Here, *β_ins_* is the biological transmission risk per insertive sex act, and *β_rec_* is the biological transmission risk per receptive sex act. The transmission risk *β’_c_* is reduced due to the condom use, with efficacy *d_c_*.

The total number of sex acts per partnership *n_sa_* is assumed to be a constant value, independent of activity level and serostatus. The proportion of *insertive* sex acts for an individual of serostatus *j*, out of total number of sex acts per partnership with a partner of serostatus *j’*, is defined as *α_j,j’_*.

***Sexual mixing: Activity groups***

There are 2 activity groups in the model: low and high, depending on the average annual number of partners *C_i_*. We assumed proportionate mixing by sexual activity (individual of activity level *i,* partner of activity level *i’*) as measured by partner change rates per year, using a previously described approach^3^.

$$\rho_{i,i^{'}}=\varepsilon\delta+\left( 1-\varepsilon\right)\frac{\sum_{j'} \left( C_{i'}I_{i',j'} \right)}{\sum_{i'} \sum_{j'} \left( C_{i'}I_{i',j'} \right)}$$

Here, *ε* = 0 (for the proportionate mixing), *δ* is the unity matrix, *C_i’_* is the number of partners per year for activity level *i’*, and *I_i’,j’_* is the compartment of the partner. The resulting 2 by 2 matrix takes the form:

$$\rho_{i,i^{'}}=\left[ \begin{matrix} \rho_{11} & \rho_{12} \\ \rho_{21} & \rho_{22} \end{matrix} \right]=\left[ \begin{matrix} \frac{P_{low}}{P_{low}+P_{high}} & \frac{P_{high}}{P_{low}+P_{high}} \\ \frac{P_{low}}{P_{low}+P_{high}} & \frac{P_{high}}{P_{low}+P_{high}} \end{matrix} \right]$$

Where the total number of partnerships *P_i_* available for each of the activity groups (*low* *i* = 1; *high* *i* = 2) is defined as:

$$P_{low}= C_{1}X_{1}+C_{1}X_{1}^{PrEP}+C_{1}I_{1}^{ND}+C_{1}I_{1}^{D}+C_{1}I_{1}^{DT}$$

$$P_{high}= C_{2}X_{2}+C_{2}X_{2}^{PrEP}+C_{2}I_{2}^{ND}+C_{2}I_{2}^{D}+C_{2}I_{2}^{DT}$$

***Sexual mixing: Serostatus***

We considered three groups by perceived serostatus, resulting in a 3 by 3 matrix: *uninfected/unknown* (*X_i_* and *I_i_^ND^*), *infected* *diagnosed with HIV* (*I_i_^D^* and *I_i_^DT^*), and *uninfected* *on PrEP* (*X_i_^PrEP^*). Before PrEP implementation, the *X_i_^PrEP^* compartment is zero, hence the mixing matrix is reduced to 2 by 2 matrix.

In the mixing matrix, the first subscript refers to the perceived serostatus of the respondent and the second refers to the perceived serostatus of the partner.

The proportionate 3 by 3 mixing matrix by serostatus can be defines as:

$p_{j,j'}=\left[ \begin{matrix} p_{11} & p_{12} & p_{13} \\ p_{21} & p_{22} & p_{23} \\ p_{31} & p_{32} & p_{33} \end{matrix} \right]$ = $\left[ \begin{matrix} \frac{P^{uninfected/unknown}}{P} & \frac{P^{diagnosed with HIV}}{P} & \frac{P^{prep}}{P} \\ \frac{P^{uninfected/unknown}}{P} & \frac{P^{diagnosed with HIV}}{P} & \frac{P^{prep}}{P} \\ \frac{P^{uninfected/unknown}}{P} & \frac{P^{diagnosed with HIV}}{P} & \frac{P^{prep}}{P} \end{matrix} \right]$

Where *P* is the total number of partnerships:

$$P= P^{uninfected/unknown}+P^{diagnosed with HIV}+P^{prep}$$

The number of partnerships per each perceived serostatus includes individuals of both activity levels, with the number of partners corresponding to each of the levels:

$$P^{uninfected/unknown}= C_{1}X_{1}+C_{1}I_{1}^{ND}+C_{2}X_{2}+C_{2}I_{2}^{ND}$$

$$P^{diagnosed with HIV}= C_{1}I_{1}^{D}+C_{1}I_{1}^{DT}+C_{2}I_{2}^{D}+C_{2}I_{2}^{DT}$$

$$P^{prep}= C_{1}I_{1}^{PrEP}+C_{2}I_{2}^{PrEP}$$

1. **Model parameters**

The estimates for model parameters are based on the IBBS 2016 study^4^ and literature sources^5-9^. The parameters are estimated for each city center separately: Yaounde and Douala and summarized in Table 1 (main text).

***Number of partnerships per year***

The number of partnerships (*C_i_*) depends on the activity class, and is estimated as a constant parameter for the low activity group, and a relative fitted parameter for the high activity group. To reflect the relationship between the sexual activity levels of the two classes, we fit the relative parameter: the ratio of the number of partners for high activity group and number of partners for the low activity group (*C_H_/C_L_*). The number of partnerships was calculated using sampling weights within each activity group based on the literature^4^.

***Proportion of high activity group***

The proportion of high activity class (N_H_/(N_H_+N_L_)) is fitted in the model, with the plausible ranges defined separately for each city separately. Activity was defined by the number of sexual partners in the past year. We used the proportion over 10 partners as the high end of the range, and the proportion over 6 partners as the low end of the range.

***ART initiation rate***

The ART initiation rate (*κ*) is fitted in the model, with the plausible ranges defined for each city separately, based on the IBBS study^4^. The rate was calculated as the number enrolled in ART treatment out of HIV diagnosed individuals, per year, for years 2012-2015 for Yaounde, and 2012-2016 for Douala. The minimum and maximum of the annual rates were accepted as a range for the model fitting.

***ART discontinuation rate***

The ART discontinuation (dropout) rate (*κ_DT_*) is a constant parameter in the model, defined for both cities, based on the IBBS study^4^. The rate was calculated as the number of discontinuations of ART treatment out of diagnosed individuals enrolled in ART, per year.

***Testing rate***

The testing rate (*ψ*) is fitted in the model, with the plausible ranges defined for each city separately, based on the IBBS study^4^. The testing rate was estimated as the proportion of individuals, who reported taking an HIV test within the last 12 months, and taking into account the fact that some individuals did not receive the test results. The testing rate based on the study overestimates the actual testing rate. However, this were the only reliable data available to us at that point.

***Transmission risk per sex act β_ins_ and β_rec_ references***

Both beta parameters were consistent for both cities. Both were defined as per-act probability of acquiring HIV from an HIV positive partner. Estimates were taken from relevant literature for β_ins_^10^ and β_rec_^11^.

***Condom efficacy d_c_ reference***

Condom efficacy was consistent for both cities and was defined as per act effectiveness. The estimate was taken from past literature^6^.

***Proportion of sex acts with condom***

The proportion of sex acts with condom (*pc*) is fitted in the model, with the plausible ranges defined for each city separately, based on the IBBS study^4^. The proportion was estimated as a weighted average for the condom use with casual and regular partners. The study includes responses for the number of sex acts with condom and with no condom for both types of partnerships (casual and regular) among individuals with negative/unknown HIV status (*pc_neg-neg_*). Among partnerships between HIV negative and HIV positive individuals, only regular partnerships are reported. Therefore, we estimated plausible ranges and fitted condom use proportion for the negative-negative type of partnerships (seroconcordant). For the negative-positive type of partnerships (serodiscordant), we estimated odds ratio (*OR*) based on the regular partnerships, and then extrapolated this odds ratio to estimate the proportion of overall condom use for the negative-positive partnerships (*pc_neg-pos_*).

To estimate the range for the condom use proportion *pc_neg-neg_*, we accepted as the lower limit the proportion of *consistent* condom use, e.g. proportion of answers ‘Always’ to the question about the frequency of condom use with a specific type of partner. The upper limit was based on the proportion of *any* condom use for the last 3 partners.

***Annual number of sex acts per partnership***

The number of sex acts per partnership per year (*n_sa_*) is a constant parameter in the model, estimated for each city separately, based on the IBBS study^4^. The estimate was based on the combination of self-reported regular partnerships within the past year and casual relationships within the past month.

***Proportion of insertive sex acts***

The proportion of insertive acts (*α*) is a constant parameter in the model. First, we estimated a weighted average of proportion of insertive acts among regular and casual partners, based on the IBBS study^4^. The estimate for the serodiscordant partnerships was in the range 0.45 to 0.55 among the respondents in both Yaounde and Douala, weighted for regular and casual partners. To simplify the analysis, we assumed equal proportion of insertive and receptive acts, independent of the serostatus of the partner and the city: *α* = 0.5.

***Proportion of virally suppressed individuals***

The proportion of virally suppressed individuals (*t_ART_* ) is a constant parameter in the model, estimated separately for Yaounde and Douala, based on the IBBS study^4^. The proportion was calculated based on the viral load tests among those who were on ART treatment reported at the time of participation, between December 2015 and October 2016. Viral suppression was considered <1000 copies/mL.

***PrEP effectiveness***

PrEP effectiveness parameter *ω* can be found in Table 1 (main text) and was based on prior literature^7^. PrEP uptake was calculated in order to establish programs that established varied PrEP coverage at the end of 20 years. The coverage rates established follow these 20-year programs were 25%, 50% and 75%.

1. **Calibration**

***Data***

The model was calibrated to the data on HIV epidemics among MSM in cities of Yaounde and Douala in Cameroon (2012-2016, IBBS^4^ and R2P studies^12,13^). Three data parameters were chosen as the calibration targets: HIV prevalence, annual number of HIV diagnoses per 10,000 MSM, and ART coverage.

*HIV prevalence*

We used city-specific MSM prevalence estimates and the associated confidence intervals from IBBS 2016 as out bound for HIV prevalence. We also used another study from 2013 to verify our range^13^.

Table A1. HIV prevalence calibration targets

|  | **Yaounde** | **Douala** | **citation** |
| --- | --- | --- | --- |
| **Prevalence 2016** | 45.1% (39.3, 51.0) | 25.9% (21.8, 31.7) | ^4^ |
| **Prevalence 2013** | 44.4% (35.7-53.2) | 25.5% (19.1-31.9) | ^12,13^ |

*Annual rate of new HIV diagnoses*

We used the diagnoses per year in the IBBS report to calculate the number of annual diagnoses per year. Time of diagnoses were available through this study and we used the sample size as the denominator for this calculation. We have reliable data several years back so we used the data between 2013 and 2016 within each city to set the high and low end of each range.

Table A2. Incident HIV diagnoses

| **Year** | **Newly diagnosed** | | **Newly diagnosed per 10,000** | | **source** |
| --- | --- | --- | --- | --- | --- |
|  | **Yaounde** | **Douala** | **Yaounde** | **Douala** |  |
| **2013** | 3 | 1 | 98 | 33 | ^4^ |
| **2014** | 9 | 3 | 294 | 98 | ^4^ |
| **2015** | 43 | 8 | 1405 | 261 | ^4^ |
| **2016** | N/A | 11 | N/A | 360 | ^4^ |
| **sample size** | 306 | 354 |  |  |  |

*ART coverage*

Using 2013-2016 data from the IBBS report, we calculated the high and low end of ART coverage based on the number of individuals on ART compared to all individuals with known positive HIV status since the end of the prior year.

Table A3. ART coverage

|  | **Yaounde** | | | **Douala** | | |  |
| --- | --- | --- | --- | --- | --- | --- | --- |
|  | **ART coverage** | **total HIV+** | **% coverage** | **ART coverage** | **total HIV+** | **% coverage** | **source** |
| **2013** | 8 | 12 | 66.67% | 12 | 21 | 57.14% | ^4^ |
| **2014** | 9 | 21 | 42.86% | 14 | 24 | 58.33% | ^4^ |
| **2015** | 37 | 64 | 57.81% | 29 | 32 | 90.63% | ^4^ |
| **2016** | N/A | N/A | N/A | 32 | 43 | 74.42% | ^4^ |

***Fitting procedure***

We calibrated the model using the baseline scenario (main text, Table 1) in two stages within a Bayesian framework. During the first stage, a Latin Hypercube sampling^14^ was used to generate 7776 (6^5^) unique parameter sets from the parameter space specified by the priors in Table 1 (main text), and acceptance-rejection method was used to select those parameter sets that satisfy the calibration constraints (Figure A2) for the following calibration targets: HIV prevalence (Table A1), the number of new HIV diagnoses rate per 10,000 MSM per year (Table A2), and ART coverage (Table A2).

Figure A2. Posterior distribution of the calibration target parameters (HIV prevalence, new HIV diagnoses per 10,000 MSM per year, ART coverage).

The calibration fits were run from the onset of epidemics up to 100 years to ensure the steady state (Figure 2, Main text). The integration was performed using Euler numerical scheme, with the time step 0.1 year. The separate runs were performed with each sampled prior set of parameters. The HIV prevalence, annual number of new diagnoses per 10,000 MSM, and the ART coverage was calculated after each run. The sets which were selected during acceptance-rejection phase are shown in Figure A3.

Figure A3. The parameter sets that produced calibration targets within the accepted limits (dark blue dots) were selected for the further analysis. The selected sets are shown as dark blue dots within the shaded box indicating the calibration limits. The epidemics produced by all sampled sets (here, only half of those sets are shown for clarity) are presented as light-colored dots.

***Fitted parameters***

The following parameters were fitted after assuming suitable distributions with plausible ranges, as indicated in the Table 1 (Main text).

1. Ratio of number of partners per year for High activity/Low activity groups.
2. Proportion of the population classified as high activity.
3. ART initiation rate per person per year.
4. Rate of HIV testing per person per year.
5. Proportion of sex acts with condom in partnerships between perceived HIV-negative / uninfected men.

The parameters are assumed to be independent, so the distribution is considered as a joint distribution of parameters.

We selected those parameter sets (using acceptance-rejection methods) that generated epidemics within the specified constraints. The second stage of the calibration comprised of using Maximum Likelihood estimates to select the best parameter sets. The model’s likelihood was calculated as a sum of log-likelihoods equally weighted for each constraint. The top 100 parameter sets resulting in the maximum cumulative log-likelihood were then chosen for the posterior parameter estimates.

***Likelihood calculations***

A log-likelihood was calculated for each parameter set as described below.

*HIV prevalence*

A binomial log-likelihood for HIV prevalence is given by:

$${LL}_{prev}=N_{prev}\left( {Data}_{prev}log\left( p_{prev} \right)+\left( 1-{Data}_{prev} \right)log\left( 1-p_{prev} \right) \right)$$

Where *N_prev_* is the sample size upon which a given observed estimate (*Data_prev_*) of MSM prevalence is based, *Data_prev_* is an observed prevalence estimate, and *p_prev_* is the modelled MSM prevalence estimate.

*ART coverage*

A binomial log-likelihood for ART coverage is given by:

${LL}_{ART}=N_{ART}\left( {Data}_{ART}log\left( p_{ART} \right)+\left( 1-{Data}_{ART} \right)log\left( 1-p_{ART} \right) \right)$

Where *N_ART_* is the sample size (the number of HIV diagnosed MSM) upon which a given observed estimate (*Data_ART_*) of ART coverage is based, *Data_ART_* is the observed ART coverage estimate, and *p_ART_* is the modelled ART coverage estimate.

*New HIV diagnoses rate*

A Poisson log-likelihood for new HIV diagnoses rate is given by:

${LL}_{DX}=I_{DX}log\left( \lambda\right)-\lambda$

Where *I_DX_* is an observed new HIV diagnoses rate estimate, per 10,000 MSM per year, and λ is the modelled estimate for the new HIV diagnoses rate, per 10,000 MSM per year.

The cumulative log-likelihood, for each successful *n*-th set of parameters, based on *a* data points for the HIV prevalence (Table A1), *b* data points for the new HIV diagnoses rate (Table A2), and *c* data points for the ART coverage (Table A3):

$${LL}_{prev\_n}=\sum_{a=1} N_{a}\left[ {Data}_{prev\_a}\log\left( {p_{prev}}_{set\_n} \right)+\left( 1-{Data}_{prev\_a} \right)log(1-{p_{prev}}_{set\_n}) \right]$$

$${LL}_{Dx\_n}=\sum_{b=1} \left( I_{{DX}_{b}}\log\left( {p_{dx}}_{set\_n} \right)-{p_{dx}}_{set\_n} \right)$$

$${LL}_{ART\_n}=\sum_{c=1} N_{c}\left[ {Data}_{ART\_c}\log\left( {p_{ART}}_{set\_n} \right)+\left( 1-{Data}_{ART\_c} \right)log(1-{p_{ART}}_{set\_n}) \right]$$

The total log-likelihood for the data given the model, for each *n-*th parameter set, is:

${LL}_{model set n}=\frac{1}{3}\left( {{LL}_{prev\_n\_norm}+LL}_{Dx\_n\_norm}+{LL}_{ART\_n\_norm} \right)$

Where the log-likelihood terms are normalized by the maximum value among calculated log-likelihoods for all parameter sets, to ensure that the values for the log-likelihood terms for all calibration targets are dimensionless and kept within 0 to 1 limit:

$${LL}_{prev\_n\_norm}=\frac{{LL}_{prev\_n}}{max\left( {LL}_{prev\_n} \right)}$$

$${LL}_{Dx\_n\_norm}=\frac{{LL}_{Dx\_n}}{max\left( {LL}_{Dx\_n} \right)}$$

$${LL}_{ART\_n\_norm}=\frac{{LL}_{ART\_i}}{max\left( {LL}_{ART\_n} \right)}$$

The results of the normalized log-likelihood calculations are shown in Figure A4. The final estimates were then obtained as the cumulative log-likelihood calculated as the equally weighted sum of the 3 normalized log-likelihood values, and the best 100 parameter sets were chosen for the final posterior estimates in the form of median and interquartile range (Table 1, Main text).

Figure A4. The MLE estimation: normalized log-likelihood for HIV prevalence, Annual new diagnoses, and ART coverage for Yaounde and Douala.

1. **References**

1. Moqueet N, Simkin A, Baral S, et al. Modeling Pre-Exposure Prophylaxis (PrEP) and the Influence of Sexual Mixing Patterns on HIV Epidemics among Men who have Sex with Men (MSM). Paper presented at: Canadian Conference on HIV/AIDS Research; 4/26/2018, 2018; Vancouver, CA.

2. Pinkerton SD, Abramson PR. The Bernoulli-process model of HIV transmission. *Handbook of economic evaluation of HIV prevention programs*: Springer; 1998:13-32.

3. Garnett GP, Anderson RM. Balancing sexual partnership in an age and activity stratified model of HIV transmission in heterosexual populations. *Mathematical Medicine and Biology: A Journal of the IMA.* 1994;11(3):161-192.

4. Health JHSoP. 2016 Integrated Biological and Behavioral Surveillance (IBBS) survey among female sex workers and men who have sex with men in Cameroon. Yaounde: Johns Hopkins Cameroon Program. 2018.

5. Baggaley RF, White RG, Boily M-C. HIV transmission risk through anal intercourse: systematic review, meta-analysis and implications for HIV prevention. *International journal of epidemiology.* 2010;39(4):1048-1063.

6. Weller S, Davis K. Condom effectiveness in reducing heterosexual HIV transmission. *Cochrane Database Syst Rev.* 2002(1):CD003255.

7. Grant RM, Lama JR, Anderson PL, et al. Preexposure chemoprophylaxis for HIV prevention in men who have sex with men. *New England Journal of Medicine.* 2010;363(27):2587-2599.

8. Cameroon Life expectancy at birth. 2017; <https://www.indexmundi.com/cameroon/life_expectancy_at_birth.html>, 2018.

9. Reniers G, Slaymaker E, Nakiyingi-Miiro J, et al. Mortality trends in the era of antiretroviral therapy: evidence from the Network for Analysing Longitudinal Population based HIV/AIDS data on Africa (ALPHA). *AIDS (London, England).* 2014;28(4):S533.

10. Patel P, Borkowf CB, Brooks JT, Lasry A, Lansky A, Mermin J. Estimating per-act HIV transmission risk: a systematic review. *Aids.* 2014;28(10):1509-1519.

11. Scott HM, Vittinghoff E, Irvin R, et al. Age, race/ethnicity, and behavioral risk factors associated with per-contact risk of HIV infection among men who have sex with men in the United States. *Journal of acquired immune deficiency syndromes (1999).* 2014;65(1):115.

12. Park JN, Papworth E, Kassegne S, et al. HIV prevalence and factors associated with HIV infection among men who have sex with men in Cameroon. *Journal of the International AIDS Society.* 2013;16(4Suppl 3).

13. Papworth E, Grosso A, Ketende S, et al. Examining risk factors for HIV and access to services among female sex workers (FSW) and men who have sex with men (MSM) in Burkina Faso, Togo and Cameroon. *Baltimore: John Hopkins University.* 2014.

14. McKay MD, Beckman RJ, Conover WJ. Comparison of three methods for selecting values of input variables in the analysis of output from a computer code. *Technometrics.* 1979;21(2):239-245.
